# Refinery 4.0, a Review of the Main Challenges of the Industry 4.0 Paradigm in Oil & Gas Downstream

**DOI:** 10.3390/s22239164

**Published:** 2022-11-25

**Authors:** Igor G. Olaizola, Marco Quartulli, Elias Unzueta, Juan I. Goicolea, Julián Flórez

**Affiliations:** 1 Vicomtech Foundation, Basque Research and Technology Alliance (BRTA), Mikeletegi 57, 20009 Donostia-San Sebastian, Spain; 2 Petronor-Repsol, San Martin 5, Edificio Muñatones, 48550 Muskiz, Spain

**Keywords:** Refinery 4.0, Oil & Gas, downstream, artificial intelligence, Industry 4.0

## Abstract

Industry 4.0 concept has become a worldwide revolution that has been mainly led by the manufacturing sector. Continuous Process Industry is part of this global trend where there are aspects of the “fourth industrial revolution” that must be adapted to the particular context and needs of big continuous processes such as oil refineries that have evolved to control paradigms supported by sector-specific technologies where big volumes of operation-driven data are continuously captured from a plethora of sensors. The introduction of Artificial Intelligence techniques can overcome the current limitations of Advanced Control Systems (mainly MPCs) by providing better performance on highly non-linear and complex systems and by operating with a broader scope in terms of signals/data and sub-systems. Moreover, the state of the art of traditional PID/MPC based solutions is showing an asymptotic improvement that requires a disruptive approach in order to reach relevant improvements in terms of efficiency, optimization, maintenance, etc. This paper shows the key aspects in oil refineries to successfully adopt Big Data and Machine Learning solutions that can significantly improve the efficiency and competitiveness of continuous processes.

## 1. Introduction

Industry 4.0 or the fourth industrial revolution has become a hype in the recent literature and is having a worldwide impact by transforming the factories into ICT (Information and Communication Technologies) enabled smart production systems [1,2,3].

The evolution of general-purpose ICT technologies leveraged by the scale economy of the global Internet market has created technological solutions that are now directly impacting how industrial processes are designed and operated.

Among the most relevant technological areas that enable Industry 4.0, we can consider the following ones [4,5,6,7]:

- IIoT (Industrial Internet of Things) [8], CPS (Cyber-physical systems) [9,10], intelligent UAV-s [11], embedded systems, soft sensors.
- High performance communications, wired and wireless [12] with 5G as the main future game-changing technology and including other key technologies such as interoperable communication standards (e.g., OPC-UA (OPC Unified Architecture (accessed on 10 October 2022) https://opcfoundation.org)) [13] or protocols such as TSN (Time-Sensitive Networking (accessed on 10 October 2022) https://1.ieee802.org/tsn/) [14,15]
- Blockchain [16,17]
- Additive Manufacturing [18]
- Virtual-Augmented Reality, Digital Twin [19,20], wearables [21], haptics [22]
- Big Data [23,24,25]
- Data Science and Artificial Intelligence [26]
- Graphics and Media Technologies [27]
- Edge, Fog, Cloud computing [28,29]
- Cybersecurity as an unavoidable requirement to enable all previous concepts [30,31]

Even if any of these technology enablers have the potential to transform industrial activities, there is a combination of them that is moving plants and factories to a cognitive revolution within the Artificial Intelligence (AI) paradigm [32,33]. According to this vision, the exploitation of a massive amount of available real-time data [34,35] will lead the current production systems to smart production [24] and process industries [36].

The Industry 4.0 concept has been created from a mainly manufacturing-centered vision. However, these concepts can be extended to other fields such as the forestry sector [37], Oil & Gas upstream [38,39,40,41], sustainability [42], and medical [43], among others. However, the analysis of downstream processes has yet to be broadly covered.

This paper aims to review the major advances in Industry 4.0 (mainly from IoT and Data Analytics perspective) and to map it to the case of oil refineries. For this purpose, we present some of the conclusions of our R&D experience in oil refineries together with the scholar’s literature in the field.

### Manufacturing vs. Continuous Processes

Thanks to the advances in AI, robotics and automation have experienced a considerable step forward in manufacturing. However, this level of automation and control is somehow state of the art in big industrial processes where efficiency and reliability requirements are even higher than in manufacturing [44].

While trends towards smart manufacturing that incorporate cutting-edge technologies are becoming one of the most paradigmatic aspects of Industry 4.0, big continuous processes such as the steel and petrochemical industry put their efforts into improving sub-processes [6]. In this sense, the steel industry includes clear examples of smart factory initiatives oriented to the applications of artificial intelligence techniques to continuous processes with high energy demand [45,46] where furnaces constitute a critical part [47] due to their energy costs and environmental impact [48].

As there are aspects of industrial manufacturing and big continuous processes such as oil refining that are notably different, the challenges and opportunities identified by the Industry 4.0 literature have to be adapted by taking into account the challenges and opportunities of the Oil & Gas sector. Yuan et al. [49] identify four main opportunities where “Smart Manufacturing” can benefit the oil refining and petrochemical industry: Operational agility, adjustable data-driven model building, abnormal situation management, and planning & scheduling for an entire oil refinery. Moreover, Monedero et al. [50] address energy consumption as one of the most relevant topics of petrochemical industries. Aligned with this proposal, we consider that AI’s main impact will be in the following three main aspects:

1. Machine Learning and Visual Analytics based process optimization (considering planning and scheduling as particular cases of processes), where Visual Analytics techniques introduce the human in the loop and allow the collaboration of human expert-knowledge and artificial intelligence.
2. Fault prediction or identification of abnormal situations as well as new means of causality analysis.
3. New knowledge/insight to experts, improving actions such as decision making, design, forensic analysis and especially orienting the human understanding within an otherwise unmanageable amount of data and set of complex models. Again, Visual Analytics techniques become fundamental to bridge the gap between Big Data/AI and humans.

#### Control, Operations, and Planning in the Oil & Gas Downstream Sector

In fact, oil refineries are already at a very high level of digitization, and since decades most processes have been fully controlled and optimized by automatic systems (PID controls, linear multivariate optimizers [51], specific deterministic optimization models for Crude Distillation Units (CDU) [52], Diesel production [53], Hydrogen network [54,55,56], etc.) both for efficiency and safety reasons. Moreover, rigorous models have been widely used for design, simulation, and planning. As Mudt. et al point out [57], the current state of the art of commercial rigorous simulation models are essential for the *a priori* setting parameters of different units and sub-process such as FCC (Fluid Catalytic Cracker), Hydrocracker, HDS (Hydrodesulfurization), production planning [58], etc. In the case of production planning, Joly et al. [59] already propose an Industry 4.0 oriented scheduling approach that benefits from technological enablers such as CPSs, wireless networks, cloud computing, and integrated Big Data. Moreover, the whole refinery-wide optimization (RWO) is also based on rigorous simulation model management software. However, all these commanding actions for planning, scheduling, and optimization still rely on the human experience [60]. With all this technology already integrated and due to the enormous CapEx costs of oil refineries, the strategies towards exploiting Data Science and Artificial Intelligence based methodologies presents challenges that must be specifically addressed [49,61].

The main goal of this paper is to show the current challenges, opportunities, and directions that can endow oil refineries with better insight and operational capabilities based on data science and AI as key enablers for the next refinery-wide cognition stage. The presented work is based in the literature review and the research activities carried out in the refinery of Petronor (http://petronor.eus/ accessed on 8 October 2022) located in the Basque Country, which is one of the five refineries of Repsol (https://www.repsol.com accessed on 8 October 2022) in Spain.

## 2. Current Operation in Refineries

### 2.1. Control, Operations, and Planning

**Figure 1:**
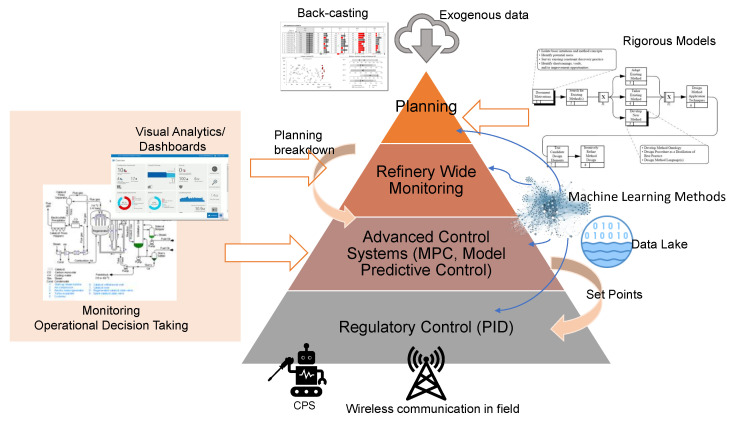
Control Pyramid endowed with data exploitation resources.

Control systems are often visualized as a pyramid [62] where higher levels mean a grouping of individual elements and a deeper of abstraction. Figure 1 represents current control, operation, and monitoring levels in a refinery. As it can be observed, many of the elements are already considered as characteristics of Industry 4.0 [63]. Automatic online optimizers that gather real-time data from along the entire plant are common practice in current refineries, and those are directly connected to information systems that allow operational decisions and planning strategies in a similar way that MES (Manufacturing Execution System) & ERP (Enterprise Resource Planning) systems are used in manufacturing [64,65].

However, the data-driven approach is still not exploited in all its potential, and control models are built on PID controllers and multivariate optimizers [57,62,66] that establish the set-points of lower-level controllers by maximizing a cost function. The parameters of these cost functions are the ones that are established in the higher levels of the pyramid. The main advantage of this approach is the good performance in terms of efficiency and robustness [67]. The drawbacks come from the lack of flexibility (it can operate only within very restricted operation regions), the need for periodical calibration (that typically requires step-tests), and the fact that no knowledge or insight is created from the huge amount of data that is continuously processed.

With the explosion of AI in industrial activities, this pyramid is being transformed by incorporating Data Science methodologies and technologies along processes. Machine Learning (ML) models are able to learn directly from historical data, creating this way knew knowledge and new perspectives of the processes that are including these techniques [68]. It is important to notice that in most cases, ML models do not substitute but enhance the currently existing control and optimization systems.

### 2.2. Integration of Machine Learning Models

**Figure 2:**
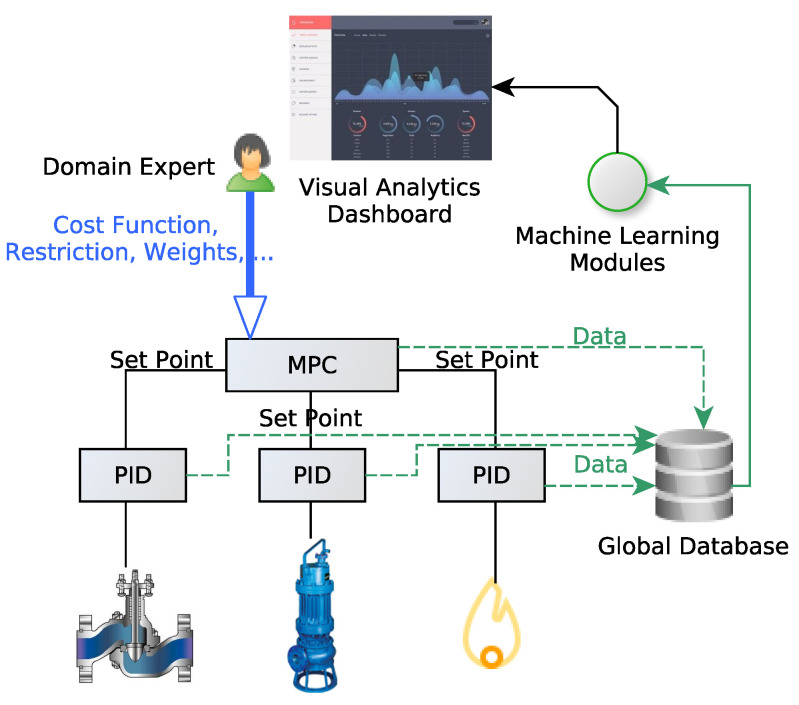
Machine Learning Modules’ Validation Process.

For the sake of safety and robustness in production efficiency, all machine learning methods have to be extensively validated before they directly command any process. Therefore, we present a validation, integration and operational testing methodology used for AI related Oil refining processes carried out within Petronor. According to this methodology, ML modules are validated first by domain experts that are already in charge of the related process. As it is depicted in Figure 2, ML modules are fed by all relevant data integrated in a global database that is able to provide real-time data (we are considering that the training and adjustment phase have already been carried out) [69]. The results of these ML modules are presented in dashboards or can be used to create a Digital Twin [70] where Visual Analytics techniques are used in order to endow the domain expert with the insight required to make the proper decisions. These decisions are then manually established on the higher-level control system (Model Predictive Control—MPC) [71]. This workflow is maintained during the whole validation period. As in most industrial to perform simultaneous A/B tests is not feasible in practice, and therefore, validation metrics consist of the comparison between periods combined with the subjective evaluation of the domain experts.

**Figure 3:**
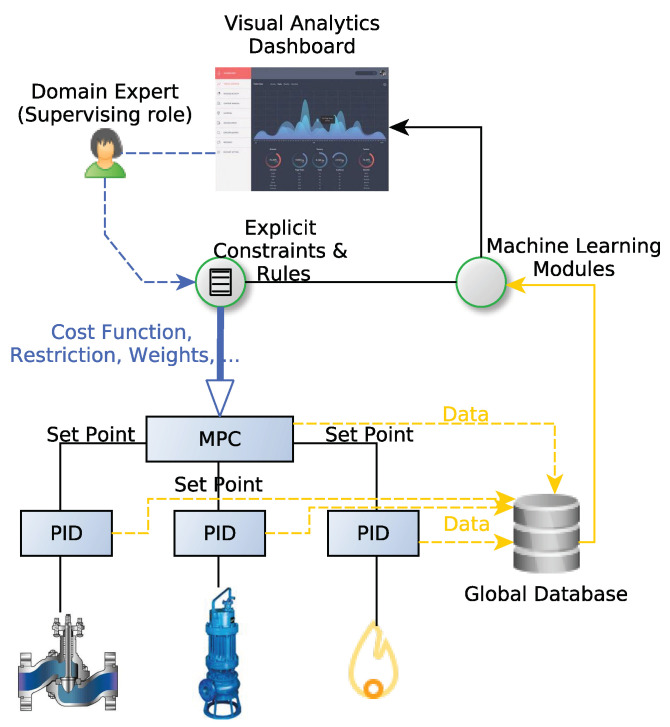
Machine Learning Modules where expert human supervising and control are introduced in the loop. MPC performs multivariate control by establishing set points on basic control systems based on previous calibrations (step tests, etc.). Machine Learning modules can identify phenomena such as model drifts and anomalies, then, suggesting modifications to MPC parameters and restrictions. Expert human operators decide if the suggested set of new parameters should be used to update the MPC.

Once the validation period is finished, if the AI system has passed the evaluation, domain expert’s actions are defined in a set of rules that will act as explicit constraints for the AI system output. The solution is gradually validated and integrated under the supervision of the domain expert (Figure 3).

## 3. Main Limitation towards Refinery 4.0

As mentioned in previous sections, the level of digitization and real-time data capturing is well established in current refineries. In the case of Petronor, more than 40,000 operation variables are recorded each minute, and other data such as chemical analytics or planning /scheduling data and maintenance-related data is stored in their corresponding databases.

### 3.1. Industrial IoT

Data availability can be considered as one of the main underlying elements to leverage Industry 4.0. IIoT and 5G communications overcome current data availability limitations in the case of big industrial process plants such as oil refineries, which are even more challenging due to their big sizes and outdoor infrastructures. The main requirements can be summarized as:

- **Ubiquity**: Most sensors and actuators of plants are based on wired communications. Hence, extensions and modifications require changes in infrastructures, increasing costs and terms.
- **Data throughput flexibility**: Data/control networks are mainly designed for reliability. Therefore, deterministic networks such as token-ring [72,73] are still used for this purpose. However, massive deployment of sensors and high and dynamic sampling rates require a technological leap where analytics services acquire data according to their specific needs. Moreover, the latency and jitter of each signal have to be dynamically controlled depending on the real-time needs of the plant.TSN and network slicing techniques [74] are required to achieve the needed QoS metrics.
- **Scalability**: Massive deployment of sensors requires high-density networks, but this circumstance is unusual in real industrial cases.
- **Reliability**: One of the main characteristics of Industrial IoT is the reliability it requires. Missing data can have a dramatic effect in prediction and control, and therefore, sensor and communication infrastructures must ensure the correct and on-time data delivery.
- **Security**: The monitoring and control network of oil refineries is the most critical communication infrastructure. Therefore, the security constraints follow the highest industry standards. Wireless communications broadly extend the risk surface and

The main barrier to the current IIoT technological availability is the lack of widespread standards [75,76] and the difficulties in guaranteeing the same level of reliability and security of currently operating wired infrastructures [77].

Besides the aforementioned issues that can hinder the development and deployment of data analysis based solutions, other challenges go beyond the technical difficulties. The main ones will be described below:

### 3.2. Data Quality: Dealing with Uncertainty

It might be obvious that data quality will be a key factor in any data-analysis related activity as Leiras et al [78] state for refinery planning use cases and Laranjeiro et al. extend for any industrial activity [79]. However, it is not trivial to determine under which conditions data quality will be good enough to produce acceptable performance metrics. In this sense, one of the main issues of dealing with existing data is that the entire data capture system deployed in refineries has been designed for control & monitoring purposes. Blake et al. [80] analyze data quality in terms of accuracy, completeness, consistency, and timeliness and conclude that data quality must be analyzed depending on the problem’s complexity. Thus, it cannot be assumed that the criteria for data capturing in plant operations coincide with the requirements for data analysis and AI.

We can distinguish two types of uncertainty:

#### 3.2.1. Epistemic Uncertainty

The aforementioned gap between data capturing criteria from operational and analysis-oriented perspectives is the cause of the epistemic uncertainty [81], where the main causes are:

- **Lack of access to relevant variables**: Variables that explain how the system is performing might not be the ones that explain how it will behave in the future (those that predictive models will need).
- **Time miss-alignment of variables**: The interdependence between physical variables (time-series) is often conditioned by a lag (e.g: propagation time). Uncertainties related to these lags can make variable relations completely unobservable.
- **State of the system**: Data observed at each moment respond to the the system’s state at this specific moment. Apart from operational data, state variables (parameters) might change due to degradation, element failures, physical modifications, etc. The uncertainty in identifying the state is the main source of conflicting data (same input variables producing different and non-compatible outputs).

#### 3.2.2. Aleatoric Uncertainty

The other source of uncertainty comes mainly from precision and sampling aspects. Again, the operational criteria to choose the sensor sampling-rate or precision are those needed to observe the process from its operational view. However, modeling requirements might be different. The sampling rate of each variable should follow the Shannon-Nyquist sampling theorem [82] and therefore will depend on the dynamic behavior of each variable. However, the individual sampling of thousands of real-time data is still a big technological issue in terms of networking and storage. Therefore, in practice, all operational data are consolidated to a general sampling rate (in the case of Petronor it corresponds to 1 minute and more than 40,000 variables). For other variables such as chemical properties measured in a laboratory, processes can last for hours or even days.

Regarding the precision, the main issue comes from the fact that uncertainty is accumulated as an unknown but additive function of precision errors of all variables. It includes precise sensors such as those for temperatures or pressures together with others that typically introduce higher errors such as flow-meters or sensors to measure the relative presence of some specific chemical component [83].

The characterization of crude-oils and especially the combined features consequence of their blending are typically based on lab condition measurements that roughly represent what is coming into the process. Moreover, the input stream can be combined with slop (Sub-products that do not meet specifications and need to be reprocessed) contributions that are totally uncharacterized.

We can then combine individual aleatoric uncertainties (Equation (1)) to define the global aleatoric uncertainty $U_{a}$, where $u_{i}$ is the uncertainty of each variable *i* and $f_{i}$ is its specific relevance in the model.

(1) $$U_{a}=\sqrt{\sum f_{i}\cdot u_{i}^{2}}$$

From a probabilistic view, we can define the uncertainty in the target variable *y* given the conditional entropy (Equation (2)) where Θ represents the epistemic uncertainty, *y* is the target value, S is the sample space and P(y) is the probability distribution of *y* [84].

(2) $$H\left(Y|U_{a},\Theta \right)=-\int_{S}\sum_{y}p\left(y|U_{a},\Theta \right)\log\left(p\left(y|U_{a},\Theta \right)\right)dP\left(s\right)$$

Hazen et al. [85] similarly describe these three main aspects of data quality for Data Science applied to supply chain management that can be generalized to industrial cases: Accuracy (aleatoric uncertainty), Timeliness Consistency, and Completeness (Epistemic Uncertainty), where completeness refers to the avoidance of missing data in existing records. In a more general industrial context, completeness should be extended to the availability of data obtained in representative conditions. This is especially relevant when datasets tend to be strongly unbalanced (predictive maintenance, failure prediction, anomaly detection).

#### 3.2.3. Data Quality Impact Cases

Oil refineries include thousands of operation variables consolidated in a historical database. Due to legacy limitations in communication infrastructures and data storage costs, typical sampling rates for storage systems are around 1 min. While this sampling period might be fast enough for many variables in a system where dynamic behavior tends to be rather slow (e.g., temperatures), there are some other phenomena that can only be observed at much higher sampling rates (e.g., perturbations in pressure measurements). Moreover, some variables—especially those related to chemical properties—are difficult to measure accurately. In the data-science projects carried out in Petronor we have concluded that in many production failure prediction problems, causes are unobservable unless sampling rates are increased.

In the specific cases of fault predictors, some of them such as extraction faults in distillation columns happen in a much smaller time span (pressure and flow changes) than the sampling periods used for control and monitoring (1 sample per minute). Under these conditions, relevant variables remain unobservable, and only indirect methods can be used. Even if these methods can predict fault risks and indirect causes, their accuracy is limited if compared with better conditions in terms of data quality (in this case, sampling period) that allow a precise prediction of when the system will fail and which are the previous conditions that lead to the failure.

### 3.3. Experimental Environment

Refineries are considered critical infrastructures and due to safety and cost reasons, the options to carry out experiments on a production system (perturbations or different operation modes, the introduction of new methods and tools, etc.) are very limited. The use of rigorous models and simulators (following the Digital Twin approach) [70,86] can mitigate this effect to some extent, but in general, testing and validation activities are always under strong constraints due to the difficulties of implementing A/B test based methodologies. In this sense, Brodersen et al. [87] propose Bayesian structural time-series models to infer causality. These experimentation constraints difficult the discovery of disruptive improvements and technology transfer [88]. In this sense, refineries are rather evolutionary than revolutionary environments that gradually adopt innovation. Thus, the main challenge of the Refinery 4.0 concept is to find a way of effectively adopting the technological advances while keeping the safety and reliability requirements during the transition.

### 3.4. Adaptability to New ICT Technologies

Proper and parallel research and experimentation ICT infrastructures that can inter-operate with plant data are necessary in order to carry out data science and AI activities that really impact plant processes and operations. However, current refineries, following an evolutionary trend, include a variety of gradually introduced complex ICT infrastructures that hardly interoperate. Moreover, the inclusion of technologies that are able to provide controlled but transparent access to the entire ICT system is a challenge that involves economic costs and creates reluctances due to potential cyber-security threats. According to a study published by Accenture in 2019 [89], 48% of refiners that participated in a survey in 2018 rated themselves as mature or semi-mature in digital technology deployment compared to 44% in 2017. This survey also shows that the main investing focus is centered on advanced process control, analytics and IoT, and cybersecurity tools.

## 4. Potential of Data Intelligence

Even if the challenges towards a data-driven refinery operation can be high, the benefits of exploiting data from a holistic view can widely outweigh these costs. Guota et al. [90] present a general study of Artificial Intelligence in the oil & gas sector, including upstream, midstream and downstream. The main downstream applications identified by these authors are related to expense reduction, production increase and improvements in maintenance and security.

### 4.1. Optimization

The classical PID + MPC control, together with the use of rigorous models for planning and engineering, is already a mature approach. It means that the efficiency of the systems designed and operated this way is near its asymptote. The inclusion of AI models that “observe” the behavior and find new optimal points (or regions) within the feature space (even out of its explored boundaries) can be a real step-change in the operation of processes where little improvements in efficiency can imply significant savings in costs (economic and environmental). Khor et al. [91] present a review work of refinery optimization approaches, concluding that while refinery-wide optimization is not feasible with the current state of the art in optimization technologies, specific optimization of aspects such as planning & scheduling, advanced process control, and product blending have been successfully addressed. While most successful methods are based on classical approaches such as MPC and linear programming, machine learning methods are becoming more popular. As an example, Tuttle et al. [92] present a method for $NO_{x}$ emission reduction based on artificial neural networks where emissions are reduced by 22.5%.

Multiobjective optimization (MOO) is a common need in a number of oil refining processes (distillation, cracking, desulfuration). Al-Jamin et al. [93] present a review of MOO methods for refinery catalytic processes.

Moreover, current advanced control systems have substantial limitations in the hierarchy levels they can deal with. It means that the global optimization of the plant cannot be managed in real time. AI systems have the potential to manage processes from a more global scope (ultimately, the entire plant).

### 4.2. Anomaly Prediction

The creation of predictive models that can identify normal behavior patterns and predict anomalies has an enormous value in plant operation. Hundi et al. [94] present a machine learning method for thermal power plants where subtle inter-dependencies between sensor data can be used to extract insights better monitoring and more reliable forecasting.

#### 4.2.1. Predictive Maintenance

Predictive maintenance in refineries implies big savings if compared with the currently applied preventive maintenance policies. On the one hand, condition-based maintenance (CBM) costs between Turnarounds (TAR) can be reduced while ensuring the reliability of the plant, and on the other hand, unexpected shutdowns can be avoided by anticipating failures [95,96]. In this sense, Digital Twin based approaches can provide failure predictions and robustness in relation to erratic behaviors [97].

Classical methods such as Linear Programming are still a valid for predictive maintenance [98] when data availability can hinder the development of machine learning models. One of the main difficulties of machine learning methods is the difficulty to create valuable datasets where anomalies are significant enough to be modeled. Helmiriawan et al. [99] present a deep learning method for maintenance in oil refineries where datasets consists of six months of normal conditions and five months of abnormal conditions. However, in general, failure prediction methods have to be trained on extremely unbalanced datasets [100,101].

#### 4.2.2. Operation Failure Prediction

Operation failure can be understood as any situation when the process outcome is out of specifications. This can be due to diverse factors such as wrong planning, deficient control, device failure, lack of quality in the input stream, etc. The effect of such failures can create slop streams, affect other processes downstream, or even force to partial or complete shutdown. Early detection or even prediction of such situations can have a relevant impact on the Overall Equipment Effectiveness (OEE) of the plant and its productivity. Machine learning methods oriented to time series such as LTSM (Long short-term memory) networks can be used to address these types of challenges [102,103].

#### 4.2.3. Causality Analysis

Machine learning methods are highly valuable tools for causality analysis (e.g., by analyzing variable importance ranks that can be directly obtained from ML methods or by making a deeper analysis of their parameters and behavior under simulated conditions). Thus, causality analysis methodologies can help to identify why processes are not performing as expected, how to avoid failure situations, and even how to re-design processes for better regular performance [87]. Interpretability methods such as LIME [104] or SHAP [105] can be used to model predictive controls such as MPCs and then interpret the effect of input variables on the control system decisions. Tissaoui et al. [106] use Shap values to interpret the results of an Xgboost predictor and thus to identify the ability of gas prices to forecast future WTI (West Texas Intermediate) crude oil prices.

### 4.3. Planning & Scheduling

Overall planning and scheduling depend not only on plant/multi-plant conditions and capabilities but on external factors such as crude-oil market, sales forecasts, finance, regulatory aspects, etc. Data science and AI can help to improve knowledge as well as to optimize or make predictions of hypothetical scenarios, empowering this way decision makers with a better insight that can provide substantial improvements in developing more effective strategies. Bueno et al. [65] show a systematic review of the literature on production planning and control, but it is mainly centered on manufacturing, and Oil & Gas process industry is not covered. On the other hand, Leiras et al. focus their research on planning in Oil refineries where uncertainty is considered, concluding that classical approaches (fuzzy programming, stochastic programming) are the most used when uncertainty has to be modeled.

Joly et al. [107] summarize the high relevance of planning in downstream and list the most challenging aspects concluding that the need for advanced multidisciplinary tools together with the deep domain experts’ knowledge is the key aspect for efficient planning. Heckl et al. [108] present a simulation-based method for fuel-transport planning in mesh like networks. Ribas et al. [109] propose a method for strategic planning model from upstream to downstream considering crude oil production, demand for refined products, and market prices as sources of uncertainty.

To the best of our knowledge, few research has been published on applying machine learning techniques on downstream planning and scheduling. Wang et al. [110] present a deep learning based approach where the price is taken as uncertainty source but demand is not considered.

## 5. Conclusions

We have presented a review of the major challenges and benefits of the Industry 4.0 revolution (especially IIoT, data-science, and AI techniques leveraged by other key enablers such as 5G and cloud/edge computing) applied to oil refineries.

Even if refineries tend to include a high level of ICT adoption, there are still substantial challenges in integrating AI solutions, managing the data quality and system uncertainty, and technologically evolving to the Refinery 4.0 concept. However, the potential benefits will be essential to the Oil & Gas sector, which will have to face a low-carbon future with a continuously restricting regulation in terms of efficiency and environmental impact.

### Main Challenges and Opportunities for Future Research

Strong challenges still have to be overcome to embrace the 4.0 paradigm. Interoperability between legacy systems and current/future data communication, processing, and storage solutions is still a big issue for the practical deployment of most data-driven initiatives. Moreover, cybersecurity aspects and, in general operational requirements of ICT infrastructures of big industrial plants do not facilitate their use in R&D experiments.

On the other hand, the inherent uncertainty sources and high complexity of downstream processes lead to situations where the current state of the art in machine learning presents serious flaws in overcoming classical approaches (DMCs, PIDs, Linear Programming) under the vast set of conditions (known as the long tail problem [111]) that such systems must face. Hence, the explainability and accountability of machine learning systems is an area of research that must to evolve for the effective deployment of AI technologies.

## References

[1] Khan A., Turowski K. A perspective on industry 4.0: From challenges to opportunities in production systems. Proceedings of the International Conference on Internet of Things and Big Data.

[2] Roblek V., Meško M., Krapež A. (2016). A Complex View of Industry 4.0. SAGE Open.

[3] Zhou K., Liu T., Zhou L. Industry 4.0: Towards future industrial opportunities and challenges. Proceedings of the 2015 12th International Conference on Fuzzy Systems and Knowledge Discovery, FSKD 2015.

[4] Wan J., Cai H., Zhou K. (2015). Industrie 4.0: Enabling technologies. Proceedings of the 2015 International Conference on Intelligent Computing and Internet of Things, ICIT 2015.

[5] Lu Y. (2017). Industry 4.0: A Survey on Technologies, Applications and Open Research Issues. J. Ind. Inf. Integr..

[6] Lu H., Guo L., Azimi M., Huang K. (2019). Oil and Gas 4.0 Era: A Systematic Review and Outlook. Comput. Ind..

[7] Ivanov D., Tang C.S., Dolgui A., Battini D., Das A. (2020). Researchers’ perspectives on Industry 4.0: Multi-disciplinary analysis and opportunities for operations management. Int. J. Prod. Res..

[8] Xu L.D., He W., Li S. (2014). Internet of Things in Industries: A Survey. IEEE Trans. Ind. Inform..

[9] Lee J., Bagheri B., Kao H.A. (2015). A Cyber-Physical Systems architecture for Industry 4.0-based manufacturing systems. Manuf. Lett..

[10] Jazdi N. (2014). Cyber physical systems in the context of Industry 4.0. Proceedings of the 2014 IEEE International Conference on Automation, Quality and Testing, Robotics, AQTR 2014.

[11] Alsamhi S.H., Shvetsov A.V., Kumar S., Hassan J., Alhartomi M.A., Shvetsova S.V., Sahal R., Hawbani A. (2022). Computing in the Sky: A Survey on Intelligent Ubiquitous Computing for UAV-Assisted 6G Networks and Industry 4.0/5.0. Drones.

[12] Li X., Li D., Wan J., Vasilakos A.V., Lai C.F., Wang S. (2017). A review of industrial wireless networks in the context of Industry 4.0. Wirel. Netw..

[13] Veichtlbauer A., Ortmayer M., Heistracher T. (2017). OPC UA integration for field devices. Proceedings of the 2017 IEEE 15th International Conference on Industrial Informatics, INDIN 2017.

[14] Time-Sensitive Networking Task Group (2017). IEEE 802.1 Time-Sensitive Networking Task Group.

[15] Zezulka F., Marcon P., Bradac Z., Arm J., Benesl T., Vesely I. (2018). Communication Systems for Industry 4.0 and the IIoT. IFAC-PapersOnLine.

[16] Viriyasitavat W., Da Xu L., Bi Z., Sapsomboon A. (2020). Blockchain-Based Business Process Management (BPM) Framework for Service Composition in Industry 4.0. J. Intell. Manuf..

[17] Tama B.A., Kweka B.J., Park Y., Rhee K.H. (2017). A critical review of blockchain and its current applications. Proceedings of the 2017 International Conference on Electrical Engineering and Computer Science: Sustaining the Cultural Heritage Toward the Smart Environment for Better Future, Palembang, Indonesia, 22–23 August 2017.

[18] Dilberoglu U.M., Gharehpapagh B., Yaman U., Dolen M. (2017). The Role of Additive Manufacturing in the Era of Industry 4.0. Procedia Manuf..

[19] Uhlemann T.H., Lehmann C., Steinhilper R. (2017). The Digital Twin: Realizing the Cyber-Physical Production System for Industry 4.0. Procedia CIRP.

[20] Parrott A., Warshaw L. (2017). Industry 4.0 and the Digital Twin.

[21] Srivastava R., Alsamhi S.H., Murray N., Devine D. (2022). Shape Memory Alloy-Based Wearables: A Review, and Conceptual Frameworks on HCI and HRI in Industry 4.0. Sensors.

[22] Contreras Masse R.A., Ochoa-Zezzatti A., García V., Mejía J., Gonzalez S. (2018). Application of IoT with haptics interface in the smart manufacturing industry. Int. J. Comb. Optim. Probl. Inform..

[23] Gokalp M.O., Kayabay K., Akyol M.A., Eren P.E., Kocyigit A. (2017). Big data for Industry 4.0: A conceptual framework. Proceedings of the 2016 International Conference on Computational Science and Computational Intelligence, CSCI 2016.

[24] Cheng Y., Chen K., Sun H., Zhang Y., Tao F. (2018). Data and knowledge mining with big data towards smart production. J. Ind. Inf. Integr..

[25] Oussous A., Benjelloun F.Z., Ait Lahcen A., Belfkih S. (2018). Big Data Technologies: A Survey.

[26] Li H., Yu H., Cao N., Tian H., Cheng S. (2020). Applications of Artificial Intelligence in Oil and Gas Development. Arch. Comput. Methods Eng..

[27] Posada J., Zorrilla M., Dominguez A., Simoes B., Eisert P., Stricker D., Rambach J., Dollner J., Guevara M. (2018). Graphics and Media Technologies for Operators in Industry 4.0. IEEE Comput. Graph. Appl..

[28] Yousefpour A., Fung C., Nguyen T., Kadiyala K., Jalali F., Niakanlahiji A., Kong J., Jue J.P. (2019). All one needs to know about fog computing and related edge computing paradigms: A complete survey. J. Syst. Archit..

[29] Naha R.K., Garg S., Georgakopoulos D., Jayaraman P.P., Gao L., Xiang Y., Ranjan R. (2018). Fog computing: Survey of trends, architectures, requirements, and research directions. IEEE Access.

[30] Lezzi M., Lazoi M., Corallo A. (2018). Cybersecurity for Industry 4.0 in the Current Literature: A Reference Framework. Comput. Ind..

[31] Kiss M., Breda G., Muha L. (2019). Information security aspects of Industry 4.0. Procedia Manuf..

[32] Li B.H., Hou B.C., Yu W.T., Lu X.B., Yang C.W. (2017). Applications of artificial intelligence in intelligent manufacturing: A review. Front. Inf. Technol. Electron. Eng..

[33] Xu L.D., Xu E.L., Li L. (2018). Industry 4.0: State of the art and future trends. Int. J. Prod. Res..

[34] Lee J., Kao H.A., Yang S. (2014). Service innovation and smart analytics for Industry 4.0 and big data environment. Procedia CIRP.

[35] Wang S., Wan J., Zhang D., Li D., Zhang C. (2016). Towards smart factory for industry 4.0: A self-organized multi-agent system with big data based feedback and coordination. Comput. Netw..

[36] Ge W., Guo L., Li J. (2017). Toward Greener and Smarter Process Industries. Engineering.

[37] Sahal R., Alsamhi S.H., Breslin J.G., Ali M.I. (2021). Industry 4.0 towards Forestry 4.0: Fire Detection Use Case. Sensors.

[38] Elijah O., Ling P.A., Rahim S.K.A., Geok T.K., Arsad A., Kadir E.A., Abdurrahman M., Junin R., Agi A., Abdulfatah M.Y. (2021). A Survey on Industry 4.0 for the Oil and Gas Industry: Upstream Sector. IEEE Access.

[39] Majstorović V. (2022). Application of Industry 4.0 model in Oil and Gas companies. J. Eng. Manag. Compet..

[40] Wanasinghe T.R., Wroblewski L., Petersen B.K., Gosine R.G., James L.A., Silva O.D., Mann G.K.I., Warrian P.J. (2020). Digital Twin for the Oil and Gas Industry: Overview, Research Trends, Opportunities, and Challenges. IEEE Access.

[41] Pandey Y.N., Rastogi A., Kainkaryam S., Bhattacharya S., Saputelli L. (2020). Toward Oil and Gas 4.0. Machine Learning in the Oil and Gas Industry.

[42] Javaid M., Haleem A., Singh R.P., Suman R., Gonzalez E.S. (2022). Understanding the adoption of Industry 4.0 technologies in improving environmental sustainability. Sustain. Oper. Comput..

[43] Javaid M., Haleem A. (2019). Industry 4.0 applications in medical field: A brief review. Curr. Med. Res. Pract..

[44] Gölzer P., Cato P., Amberg M. Data Processing Requirements of Industry 4.0—Use Cases for Big Data Applications. Proceedings of the ECIS 2015 Research-in-Progress Papers.

[45] Dettori S., Matino I., Colla V., Weber V., Salame S. (2019). Neural network-based modeling methodologies for energy transformation equipment in integrated steelworks processes. Energy Procedia.

[46] Colla V., Matino I., Dettori S., Cateni S., Matino R. (2019). Reservoir computing approaches applied to energy management in industry. Commun. Comput. Inf. Sci..

[47] Matino I., Dettori S., Colla V., Weber V., Salame S. (2019). Forecasting blast furnace gas production and demand through echo state neural network-based models: Pave the way to off-gas optimized management. Appl. Energy.

[48] Filipponi M., Rossi F., Presciutti A., De Ciantis S., Castellani B., Carpinelli A. (2016). Thermal analysis of an industrial furnace. Energies.

[49] Yuan Z., Qin W., Zhao J. (2017). Smart Manufacturing for the Oil Refining and Petrochemical Industry. Engineering.

[50] Monedero I., Biscarri F., León C., Guerrero J.I., González R., Pérez-Lombard L. (2012). Decision system based on neural networks to optimize the energy efficiency of a petrochemical plant. Expert Syst. Appl..

[51] Carroll J.A., Horne R.N. Multivariate optimization of production systems. Proceedings of the SPE Annual Technical Conference and Exhibition.

[52] Alattas A.M., Grossmann I.E., Palou-Rivera I. (2011). Integration of nonlinear crude distillation unit models in refinery planning optimization. Ind. Eng. Chem. Res..

[53] Garcia M.R., Pitta R.N., Fischer G.G., Neto E.R. (2014). Optimizing diesel production using advanced process control and dynamic simulation. IFAC Proceedings Volumes (IFAC-PapersOnline).

[54] Fonseca A., Sá V., Bento H., Tavares M.L., Pinto G., Gomes L.A. (2008). Hydrogen distribution network optimization: A refinery case study. J. Clean. Prod..

[55] Lou J., Liao Z., Jiang B., Wang J., Yang Y. (2014). Robust optimization of hydrogen network. Int. J. Hydrogen Energy.

[56] Sardashti Birjandi M.R., Shahraki F., Birjandi M.S., Nobandegani M.S. (2014). Application of global optimization strategies to refinery hydrogen network. Int. J. Hydrogen Energy.

[57] Mudt D.R., Pedersen C.C., Jett M.D., Karur S., McIntyre B., Robinson P.R. (2007). Refinery-Wide Optimization with Rigorous Models. Practical Advances in Petroleum Processing.

[58] Neiro S.M., Pinto J.M. (2005). Multiperiod optimization for production planning of petroleum refineries. Chem. Eng. Commun..

[59] Joly M., Odloak D., Miyake M., Menezes B.C., Kelly J.D. (2018). Refinery production scheduling toward Industry 4.0. Front. Eng. Manag..

[60] Qian F., Zhong W., Du W. (2017). Fundamental Theories and Key Technologies for Smart and Optimal Manufacturing in the Process Industry. Engineering.

[61] Pandey A., Branson D. (2020). 2020 Digital Operations study for energy, Oil and Gas.

[62] Campos M., Teixeira H., Liporace F., Gomes M. (2009). Challenges and problems with advanced control and optimization technologies. IFAC Proceedings Volumes (IFAC-PapersOnline).

[63] Rojko A. (2017). Industry 4.0 concept: Background and overview. Int. J. Interact. Mob. Technol..

[64] Mantravadi S., Møller C. (2019). An overview of next-generation manufacturing execution systems: How important is MES for industry 4.0?. Procedia Manuf..

[65] Bueno A., Godinho Filho M., Frank A.G. (2020). Smart production planning and control in the Industry 4.0 context: A systematic literature review. Comput. Ind. Eng..

[66] Parkash S. (2003). Refinery Linear Programming Modeling. Refining Processes Handbook.

[67] Geng Z., Zhang Y., Li C., Han Y., Cui Y., Yu B. (2020). Energy optimization and prediction modeling of petrochemical industries: An improved convolutional neural network based on cross-feature. Energy.

[68] Keller M., Rosenberg M., Brettel M., Friederichsen N. (2014). How Virtualization, Decentrazliation and Network Building Change the Manufacturing Landscape: An Industry 4.0 Perspective. Int. J. Mech. Aerospace, Ind. Mechatron. Manuf. Eng..

[69] Mohammadpoor M., Torabi F. (2019). Big Data analytics in oil and gas industry: An emerging trend. Petroleum.

[70] Min Q., Lu Y., Liu Z., Su C., Wang B. (2019). Machine Learning based Digital Twin Framework for Production Optimization in Petrochemical Industry. Int. J. Inf. Manag..

[71] Maxim A., Copot D., Copot C., Ionescu C.M. (2019). The 5w’s for control as part of industry 4.0: Why, what, where, who, and when—A PID and MPC control perspective. Inventions.

[72] Pitt D. (1987). Standards for the token ring. IEEE Netw..

[73] Follows J. (2000). Token Ring Solutions.

[74] Wu Y., Dai H.N., Wang H., Xiong Z., Guo S. (2022). A Survey of Intelligent Network Slicing Management for Industrial IoT: Integrated Approaches for Smart Transportation, Smart Energy, and Smart Factory. IEEE Commun. Surv. Tutorials.

[75] Zawra L.M., Mansour H.A., Eldin A.T., Messiha N.W. (2017). Utilizing the Internet of Things (IoT) Technologies in the Implementation of Industry 4.0. Proceedings of the International Conference on Advanced Intelligent Systems and Informatics 2017.

[76] Aziz A., Schelén O., Bodin U. (2020). A Study on Industrial IoT for the Mining Industry: Synthesized Architecture and Open Research Directions. IoT.

[77] Parks R.C., Rogers E. (2008). Vulnerability Assessment for Critical Infrastructure Control Systems. IEEE Secur. Priv. Mag..

[78] Leiras A., Ribas G., Hamacher S., Elkamel A. (2011). Literature review of oil refineries planning under uncertainty. Int. J. Oil Gas Coal Technol..

[79] Laranjeiro N., Soydemir S.N., Bernardino J. A Survey on Data Quality: Classifying Poor Data. Proceedings of the 2015 IEEE 21st Pacific Rim International Symposium on Dependable Computing, PRDC 2015.

[80] Blake R., Mangiameli P. (2011). The effects and interactions of data quality and problem complexity on classification. J. Data Inf. Qual..

[81] Kiureghian A.D., Ditlevsen O. (2009). Aleatory or epistemic? Does it matter?. Struct. Saf..

[82] Jerri A.J. (1977). The Shannon Sampling Theorem—Its Various Extensions and Applications: A Tutorial Review. Proc. IEEE.

[83] Khodabakhsh A., Ari I., Bakir M., Ercan A.O. (2018). Multivariate Sensor Data Analysis for Oil Refineries and Multi-mode Identification of System Behavior in Real-time. IEEE Access.

[84] Feder M., Merhav N. (1994). Relations Between Entropy and Error Probability. IEEE Trans. Inf. Theory.

[85] Hazen B.T., Boone C.A., Ezell J.D., Jones-Farmer L.A. (2014). Data quality for data science, predictive analytics, and big data in supply chain management: An introduction to the problem and suggestions for research and applications. Int. J. Prod. Econ..

[86] Ahsan M. (2015). Prediction of gasoline yield in a fluid catalytic cracking (FCC) riser using k-epsilon turbulence and 4-lump kinetic models: A computational fluid dynamics (CFD) approach. J. King Saud Univ. Eng. Sci..

[87] Brodersen K.H., Gallusser F., Koehler J., Remy N., Scott S.L. (2015). Inferring causal impact using bayesian structural time-series models. Ann. Appl. Stat..

[88] Mousaei A. (2018). Designing a Specific Model for Technology Transfer in Oil, Gas, and Petrochemical Sectors.

[89] Tracey C., Richard H., Andy C., Elfije L., Julie A. (2019). The Intelligent Refinery. Technical Report.

[90] Gupta D., Shah M. (2021). A comprehensive study on artificial intelligence in oil and gas sector. Environ. Sci. Pollut. Res..

[91] Khor C.S., Varvarezos D. (2016). Petroleum refinery optimization. Optim. Eng..

[92] Tuttle J.F., Vesel R., Alagarsamy S., Blackburn L.D., Powell K. (2019). Sustainable NOx emission reduction at a coal-fired power station through the use of online neural network modeling and particle swarm optimization. Control. Eng. Pract..

[93] Al-Jamimi H.A., BinMakhashen G.M., Deb K., Saleh T.A. (2021). Multiobjective optimization and analysis of petroleum refinery catalytic processes: A review. Fuel.

[94] Hundi P., Shahsavari R. (2020). Comparative studies among machine learning models for performance estimation and health monitoring of thermal power plants. Appl. Energy.

[95] Antomarioni S., Pisacane O., Potena D., Bevilacqua M., Ciarapica F.E., Diamantini C. (2019). A predictive association rule-based maintenance policy to minimize the probability of breakages: Application to an oil refinery. Int. J. Adv. Manuf. Technol..

[96] Antomarioni S., Bevilacqua M., Potena D., Diamantini C. (2019). Defining a data-driven maintenance policy: An application to an oil refinery plant. Int. J. Qual. Reliab. Manag..

[97] Sahal R., Alsamhi S.H., Breslin J.G., Brown K.N., Ali M.I. (2021). Digital Twins Collaboration for Automatic Erratic Operational Data Detection in Industry 4.0. Appl. Sci..

[98] Pisacane O., Potena D., Antomarioni S., Bevilacqua M., Ciarapica F.E., Diamantini C. (2020). Data-driven predictive maintenance policy based on multi-objective optimization approaches for the component repairing problem. Eng. Optim..

[99] Helmiriawan H., Al-Ars Z. (2019). Multi-target Regression Approach for Predictive Maintenance in Oil Refineries Using Deep Learning. Int. J. Neural Netw. Adv. Appl..

[100] Ren Y. (2021). Optimizing Predictive Maintenance With Machine Learning for Reliability Improvement. ASCE-ASME J. Risk Uncert Engrg. Sys. Part B Mech. Engrg..

[101] Dangut M.D., Skaf Z., Jennions I.K. (2022). Handling imbalanced data for aircraft predictive maintenance using the BACHE algorithm. Appl. Soft Comput..

[102] Han Y., Ding N., Geng Z., Wang Z., Chu C. (2020). An optimized long short-term memory network based fault diagnosis model for chemical processes. J. Process. Control..

[103] Shao B., Hu X., Bian G., Zhao Y. (2019). A Multichannel LSTM-CNN Method for Fault Diagnosis of Chemical Process. Math. Probl. Eng..

[104] Zafar M.R., Khan N. (2021). Deterministic Local Interpretable Model-Agnostic Explanations for Stable Explainability. Mach. Learn. Knowl. Extr..

[105] den Broeck G.V., Lykov A., Schleich M., Suciu D. (2022). On the Tractability of SHAP Explanations. J. Artif. Intell. Res..

[106] Tissaoui K., Zaghdoudi T., Hakimi A., Nsaibi M. (2022). Do Gas Price and Uncertainty Indices Forecast Crude Oil Prices? Fresh Evidence Through XGBoost Modeling. Comput. Econ..

[107] Joly M. (2012). Refinery production planning and scheduling: The refining core business. Braz. J. Chem. Eng..

[108] Heckl I., Borbás P.I., Szombathelyi B., Frits M. (2015). Simulator for Distribution Scheduling in Downstream. MACRo 2015.

[109] Ribas G.P., Hamacher S., Street A. (2010). Optimization under uncertainty of the integrated oil supply chain using stochastic and robust programming. Int. Trans. Oper. Res..

[110] Wang C., Peng X., Shang C., Fan C., Zhao L., Zhong W. (2021). A deep learning-based robust optimization approach for refinery planning under uncertainty. Comput. Chem. Eng..

[111] Mitchell M. (2019). Artificial Intelligence: A Guide for Thinking Humans.

